# Emerging Therapeutic Targets for Spontaneous Intracerebral Hemorrhage

**DOI:** 10.2174/011570159X390388250708063345

**Published:** 2025-07-11

**Authors:** Alessandro Pezzini

**Affiliations:** 1 Department of Medicine and Surgery, University of Parma, Parma, Italy;; 2 Stroke Care Program, Department of Emergency, Parma University Hospital, Parma, Italy

## 1 INTRODUCTION

Intracerebral hemorrhage (ICH), the most common stroke subtype after ischemic stroke, takes place when a blood vessel within the brain breaks down [1]. In ~20% of cases, this can be a complication of pre-existing macrovascular causes, including arteriovenous malformations and fistulas, aneurysms, cavernous malformations, or other, rarer, underlying causes, such as reversible cerebral vasoconstriction syndrome, moyamoya disease, vasculitis, and cerebral venous sinus thrombosis. In ~80% of cases, ICH occurs without any underlying lesion (spontaneous ICH, sICH), as result of a degenerative cerebral small vessel disease, mainly cerebral amyloid angiopathy (CAA), a cerebral vascular disease characterized by increased vascular fragility due to the deposition of β-amyloid on the vessel wall and arteriolosclerosis (historically referred to as “hypertensive arteriopathy” or, more properly, deep perforator arteriopathy). According to the Global Burden of Disease Study 2019, ICH affects ~3.5 million people per year, accounting for 27.9% of all incident strokes worldwide, is the 4^th^ leading cause of premature death and is responsible for an estimated ~70 million years of life lost owing to premature mortality and years lived with disability (DALYs) [1]. A reason for this is that, despite ongoing research, effective treatments for acute sICH remain limited to anticoagulation reversal, blood pressure management, serum glucose control, and minimally invasive surgical removal of the hematoma in highly selected cases, with little individual beanefit. Notwithstanding, over the past few years, some promising targets that may reduce secondary injury after cerebral bleeding have been investigated in the setting of clinical trials and deserve consideration because of their potential clinical impact. They include 1) endogenous absorption of hematoma, 2) inhibition of iron toxicity (ferroptosis) from blood components, 3) modulation of ICH-related inflammatory responses, and 4) attenuation of perihematomal edema (PHE).

## 2 ENDOGENOUS ABSORPTION OF THE HEMATOMA

Surgical removal of the hematoma has been shown to be effective only in a highly selected subgroup of sICH patients, specifically those with a lobar hemorrhage and within the early time window [2]. Therefore, approaches aimed at promoting and accelerating endogenous resorption of bleeding and, consequently, of hemolytic blood products, can potentially reduce secondary brain injury following sICH. Erythrophagocytosis is the process by which microglia and monocyte-derived macrophages (Mϕ), recruited to the site of bleeding, phagocytose red blood cells, contributing to hematoma resolution [3]. However, this endogenous erythrophagocytosis is largely insufficient to remove hemolytic products and halt the resulting brain damage, with obvious consequences for the patient’s recovery. Thus, potentiation of endogenous erythrophagocytosis may be a crucial treatment option in case of cerebral bleeding. The scavenger receptors in macrophages (Mϕ), which are crucial for cell-to-cell interactions during the early stages of erythrophagocytosis, are regulated by liver X receptors (LXR) and peroxisome proliferator-activated receptors (PPARs), including the α, β/delta, and Ƴ isotypes [4]. Among the latter, PPAR-Ƴ is the regulator for which the most data are available. PPAR-Ƴ promotes erythrophagocytosis by upregulating the scavenger receptors. Expression of scavenger receptor genes enhances PPAR-Ƴ signaling, a mechanism which has been found to increase hematoma resorption in experimental settings [5]. Notwithstanding, a study “Safety of Pioglitazone for Hematoma Resolution in Intracerebral Hemorrhage” (SHRINC; NCT00827892), the only phase II clinical trial conducted so far in which the potent PPAR-Ƴ agonist pioglitazone was tested in patients with sICH, showed no detectable effect of this agent on patients’ mortality, cerebral edema, or functional outcomes [6]. It should be noted, however, that the number of patients included in the trial (n = 84) is likely too small to detect a difference in hard clinical outcomes between the treatment group and the placebo group.

## 3 IRON TOXICITY

In case of cerebral hemorrhage, hemoglobin (Hb) is released from lysed red blood cells, thus increasing the iron concentrations in the brain tissue [7]. Toxins of Hb are phagocytosed by microglia and nearby infiltrating macrophages, and these toxins are metabolized into iron, which indirectly increases the iron level. If there is too much iron, microglia and macrophages may be able to process it only partially, which leads to uptake of excess iron by surrounding neurons and astrocytes, increased reactive oxygen species (ROS) production within these cells, and eventually, ferroptosis, a specific mechanism of cell death. The phase II clinical trial “Intracerebral Hemorrhage Deferoxamine” (i-DEF; NCT02175225) investigated the effect of the iron chelator agent deferoxamine mesylate infused within 24 hours of sICH onset on 90-day functional outcomes. Although the agent had a negligible effect on short-term outcomes [8], it was found to accelerate 6-month recovery and long-term functional outcomes in a post hoc analysis of trial data, with no major safety concerns [9].

## 4 MODULATION OF INFLAMMATORY RESPONSES

Neuroinflammation results from the activation of several pathways surrounding the hematoma at different time points. Due to its non-antimicrobial properties, including inhibition of pro-inflammatory microglia/macrophages and matrix metalloproteases (MMPs) as well as antioxidant effects, minocycline, a second-generation tetracycline, has been investigated in two small phase I/II trials, “Minocycline in Acute Cerebral Hemorrhage” (MACH; NCT01805895) [10] and “Minocycline and Matrix Metalloproteinase Inhibition in Acute Intracerebral Hemorrhage” (MITCH; NCT03040128) [11]. Minocycline proved effective in reducing serum MMP-9 levels in patients with sICH but had no impact on patients’ clinical outcomes. Interestingly, the agent was found to be associated with reduced risk of sICH recurrence in patients with CAA, a finding that substantiates the ongoing phase I/II study “Antibiotics Against Amyloid Angiopathy” (BATMAN; NCT05680389) [12]. Likewise, the reduction in IL-6 serum levels in the sub-group of sICH patients receiving anakinra, a recombinant human IL-1 receptor antagonist and functional regulator of microglia, in a phase II study, titled “Blocking the Cytokine IL-1 in ICH” (BLOC-ICH; NCT03737344) [13], is the biological underpinning of the phase II trial “Studying Anakinra to Reduce Secondary Brain Damage After Spontaneous Hemorrhagic Stroke” (ACTION; NCT04834388) [14] currently underway. Finally, the effects of the two agonists of the sphingosine-1-phosphate (S1P) receptors, fingolimod and siponimod, were investigated because of their anti-inflammatory properties and regulatory functions in the microglia. The former agent was tested in the phase I study “Fingolimod as a Treatment of Cerebral Edema After Intracerebral Hemorrhage” (FITCH, NCT04088630), whose results are pending [15], and the latter in the phase II trial “Efficacy, Safety and Tolerability of BAF312 Compared to Placebo in Patients with Intracerebral Hemorrhage” (NCT03338998) that was prematurely halted because of the COVID-19 outbreak [16].

## 5 ATTENUATION OF PHE

In a rat model, glibenclamide (glyburide), a sulfonylurea, was found to protect blood-brain-barrier (BBB) integrity and improve neurological outcomes after ICH by inhibiting the Sur1-Trpm4 channel, leading to reduced expression of MMPs, increased levels of BBB tight-junction proteins, and reduced PHE [17]. Conversely, in the subsequent phase II trial “Glibenclamide Advantage in Treating Edema after Intracerebral Hemorrhage” (GATE-ICH; NCT03741530), glibenclamide did not provide any functional improvement at 3 months and was associated with a higher risk of hypoglycemia [18]. Reasons for the negative results of primary outcome might be attributed to relatively small haematoma volume in the patients enrolled in the GATE-ICH trial, which usually resulted in milder neurologic deficits and more favourable clinical outcomes and to the long time frame between symptom onset and enrollment (approximately 72 hours), which resulted in edema formation in some patients before the initiation of glibenclamide treatment.

Following the initial observation that celecoxib, a selective cyclo-oxygenase 2 (COX2) inhibitor, reduces PHE in animal models [19], a pilot study “Administration of Celecoxib for Treatment of Intracerebral Hemorrhage” (ACE-ICH; NCT00526214) was conducted, which indicated that treating patients within 24 hours of cerebral bleeding is associated with reduced PHE expansion [20]. The effects of administering celecoxib within 6 hours of the onset of ICH are currently being investigated in a phase II clinical trial (NCT05434065). Finally, whether the anti-inflammatory properties and the effect in decreasing BBB permeability of statins have any influence on ICH outcome and whether such an influence might depend on the APOE genotype [21] are being explored in the phase III study “Statin Use in Intracerebral Hemorrhage Patients” (SATURN; NCT03936361) [22] and the phase II/III trial “Statins for Neuroprotection in Spontaneous Intracerebral Hemorrhage” (STATIC; NCT04857632) [23].

## 6 POTENTIAL NEW TARGETS

In addition to the targets reported above, others have shown promise in animal models and are potentially suitable for future investigation in clinical trials. These include, among the others 1) downregulation of the 18 kDa translocator protein (TSPO; potential drug, etifoxine [24]), 2) inhibition of the complement system, a part of the innate immune response that enhances the ability of antibodies and phagocytic cells to clear pathogens and damaged cells (potential drug, N-acetyl heparin [NAH]) [25], 3) upregulation and modulation of aquaporin 4 (AQP4)-mediated signals, which may involve several mechanisms, including transcriptional up-regulation of AQP4 through the activation of the p38 mitogen activated protein kinase (MAPK) pathway during oxidative stress, up-regulation of AQP4 through the phosphatidylinositol 3 kinase (PI3K) signaling pathway-dependent rearrangement of actin microfilaments in response to oxidative stress and the astrocyte expression of AQP-4 induced by the Janus kinase/signal transducer and activator of transcription (JAK/STAT) signaling pathway (potential drug, edaravone) [26], 4) antagonization of the pro-inflammatory effect of IL-15 and potentiation of the anti-inflammatory IL-33 and IL-27-mediated signals [27-29], 5) reduction of the neurological deficit through activation of the programmed death ligand 1 (PD-L1) pathway [30]. For many of these potential therapeutic strategies, data are not yet available except from animal models, and more clinical evidence will, therefore, be necessary before any conclusions can be drawn regarding their applicability in treating ICH patients. What appears obvious as of now is that translating these experimental findings into clinical practice will require exploiting the cumulative effect of complementary therapeutic approaches, acting at different time-points on different targets and modulating different biological mechanisms. It is conceivable that, in the near future, treatments for which benefits have already been proven will be combined with new evidence-based acute intervention strategies (Table **1**).

## CONCLUSION

Although the identification of potential new treatment options for sICH remains a challenge, advancements have been made over the last years in understanding the complex mechanisms of bleeding-related cerebral injury, promising therapeutic targets are emerging, and the time has come to advance toward large-scale clinical trials. Based on the above findings, clinicians should reconsider their approach to ICH, which should be grounded in a multidisciplinary perspective and envision a reorganization of care systems.

## Figures and Tables

**Table 1:** Summary of potential therapeutic targets for intracerebral hemorrhage.

**Drug**	**Study**	**Trial Number**	**Reference No.**	**No. of Patients Enrolled**	**Study Design**	**Outcome**
Pioglitazone	Safety of Pioglitazone for Hematoma Resolution in Intracerebral Hemorrhage (SHRINC)	NCT00827892	[6]	84	Phase II; randomized, double-blind, placebo controlled	No effect on mortality, cerebral edema, and functional outcomes
Deferoxamine	Intracerebral Hemorrhage Deferoxamine (i-DEF)	NCT02175225	[8, 9]	291	Phase II; randomized, double-blind, placebo controlled	No functional improvement at day 90, but drug accelerated recovery up to 6 months
Minocycline	Minocycline in Acute Cerebral Hemorrhage (MACH)	NCT01805895	[10]	16	Phase I/II; randomized, open-label, evaluator blind	No effect on functional outcome, level of inflammatory biomarkers, hematoma volume, or PHE at 90 days
	Minocycline and Matrix Metalloproteinase Inhibition in Acute Intracerebral Hemorrhage (MITCH)	NCT03040128	[11]	20	Phase II; randomized, double-blind, placebo controlled	No effect on clinical outcome, but drug decreased MMP-9 level
	Antibiotics Against Amyloid Angiopathy (BATMAN)	NCT05680389	[12]	-	Phase II; randomized, double-blind, placebo controlled	Currently recruiting
Anakinra	Blocking the Cytokine IL-1 in ICH (BLOC-ICH)	NCT03737344	[13]	25	Phase II; randomized, double-blind, placebo controlled	No effect on edema and functional outcome at 3 months, but drug reduced IL-6 levels
	Anakinra to Reduce Secondary Brain Damage After Spontaneous Hemorrhagic Stroke (ACTION)	NCT04834388	[14]	75	Phase II; randomized, open-label, placebo controlled	Currently recruiting
Fingolimod	Fingolimod as a Treatment of Cerebral Edema After Intracerebral Hemorrhage (FITCH)	NCT04088630	[15]	28	Phase I; randomized, double-blind, placebo controlled	Pending
Siponimod	Efficacy, Safety and Tolerability of BAF312 Compared to Placebo in Patients With Intracerebral Hemorrhage	NCT03338998	[16]	32	Phase II; randomized, double-blind, placebo controlled	No effect on PHE volume
Glibenclamide	Glibenclamide Advantage in Treating Edema After Intracerebral Hemorrhage (GATE-ICH)	NCT03741530	[18]	200	Phase II; randomized, open-label, evaluator-blind	Currently recruiting
Colecoxib	Administration of Celecoxib for Treatment of Intracerebral Hemorrhage (ACE-ICH)	NCT00526214	[20]	60	Phase II; randomized, open-label	Currently recruiting
Statins	Statin Use in Intracerebral Hemorrhage Patients (SATURN)	NCT03936361	[22]	1456	Phase III; randomized, open-label, evaluator-blind	Currently recruiting
Atorvastatin	Statins for Neuroprotection in Spontaneous Intracerebral Hemorrhage (STATIC)	NCT04857632	[23]	98	Phase II/ III; randomized, open-label, evaluator-blind	Currently recruiting

## LIST OF ABBREVIATIONS

LXR: Liver X Receptors
PHE: Perihematomal Edema
sICH: Spontaneous ICH
CAA: Cerebral Amyloid Angiopathy
ICH: Intracerebral Hemorrhage
